# The Self-Fulfilling Prophecy of Episodic Memory Impairment in Mild Cognitive Impairment: Do Episodic Memory Deficits Identified at Classification Remain Evident When Later Examined with Different Memory Tests?

**DOI:** 10.1155/2013/437013

**Published:** 2013-08-26

**Authors:** Shannon Zofia Klekociuk, Mathew James Summers

**Affiliations:** ^1^School of Psychology, University of Tasmania, Launceston, TAS 7250, Australia; ^2^Wicking Dementia Research & Education Centre, School of Medicine, University of Tasmania, TAS, Australia

## Abstract

Previous studies of mild cognitive impairment (MCI) have been criticised for using the same battery of neuropsychological tests during classification and longitudinal followup. The key concern is that there is a potential circularity when the same tests are used to identify MCI and then subsequently monitor change in function over time. The aim of the present study was to examine the evidence of this potential circularity problem. The present study assessed the memory function of 72 MCI participants and 50 healthy controls using an alternate battery of visual and verbal episodic memory tests 9 months following initial comprehensive screening assessment and MCI classification. Individuals who were classified as multiple-domain amnestic MCI (a-MCI+) at screening show a significantly reduced performance in visual and verbal memory function at followup using a completely different battery of valid and reliable tests. Consistent with their initial classification, those identified as nonamnestic MCI (na-MCI) or control at screening demonstrated the highest performance across the memory tasks. The results of the present study indicate that persistent memory deficits remain evident in amnestic MCI subgroups using alternate memory tests, suggesting that the concerns regarding potential circularity of logic may be overstated in MCI research.

## 1. Introduction

The concept of Mild Cognitive Impairment (MCI) emerged from a series of MAYO clinic epidemiological studies attempting to identify predictive risk factors for Alzheimer's dementia (AD) [1–3]. The utility of MCI was perceived to be its ability to identify individuals most at risk of future cognitive decline, particularly those likely to transition to AD [2]. Subsequently, the clinical features used to classify MCI have gradually been replaced by MCI diagnostic criteria [4, 5], although a number of researchers question whether these criteria lack appropriate sensitivity and specificity to be considered diagnostic [6–12]. Current MCI classification criteria include concern regarding a change in cognitive functioning; evidence of objective dysfunction (usually from neuropsychological assessment); relatively intact daily functioning; and an absence of dementia [4]. According to the diagnostic criteria outlined by Winblad et al. [5], amnestic subtypes are defined by the presence of an episodic memory deficit, whereas non-amnestic subtypes are defined by the presence of a non-memory deficit (e.g., attention, language, working memory). Both of these broad variants may be further classified as single domain (deficits are limited to one cognitive domain, e.g., episodic memory) or multiple domain (deficits are present in more than one domain, e.g., memory and attention) [5]. 

The aim of many MCI studies is to follow an MCI cohort over time to identify the most sensitive predictors of future cognitive decline. Some of these studies classify patients with MCI and monitor cognitive function over time using the same battery of neuropsychological tests (e.g., [13–15]). This has introduced a concern regarding the independence of the assessment of cognitive function over time from the initial diagnosis/classification of MCI. Specifically, it raises the question as to whether individuals who maintain a specific MCI classification at follow up do so because of genuine neuropsychological impairment or because of the self-fulfilling prophecy created from using the same psychometric instruments to identify MCI [16, 17]. Other studies attempt to avoid this issue by using broad screening measures (e.g., MMSE, CERAD) to classify MCI and then track the progression of MCI cohorts using tests of discreet neuropsychological functions (e.g., [18, 19]). However, this introduces an alternative issue regarding the accuracy of the initial MCI classification. Research has revealed that broad screening measures lack the sensitivity to detect non-memory deficits (e.g., attention, language, working memory) in MCI, based on evidence that a majority of MCI cases demonstrate such deficits when assessed using reliable and valid neuropsychological measures [6, 13, 17, 20–22]. In attempt to avoid circularity, previous studies utilising restricted screening protocols may have misclassified MCI and/or missed classifying genuine cases of MCI.

The extent to which circular reasoning is an issue for the assessment of MCI remains unclear. One way of reducing its potential effects is by using a separate test battery to classify MCI, and an alternative test battery to assess cognitive function over time [23]. The present study represents an exploration into the potential issue of circular logic by examining memory function in an MCI cohort. We attempted to investigate whether amnestic dysfunction remained evident when groups were assessed using alternate tests of visual and verbal memory at screening and follow up. It was hypothesised that if circularity of logic affects MCI classification, then MCI subtypes would display a change in their memory performance across two independent neuropsychological batteries. 

## 2. Method

### 2.1. Study Population

Community-residing older adults from Tasmania (Australia) were recruited using consecutive sampling from advertisements placed in local media (TV and radio) and local general medical practices. Participants were recruited to participate in a larger longitudinal study tracking the neuropsychological profile of MCI subtypes. Each participant provided fully informed consent prior to the commencement of the study, in accordance with the Human Research Ethics Committee (Tasmania) Network and National Health and Medical Research Council (NHMRC) of Australia Human Research Guidelines, in accordance with the Declaration of Helsinki (1964).

Each participant underwent pre-screening via telephone to ensure that there were no medical, neurological, or psychological conditions that would impact their participation. In addition, each participant who passed pre-screening was assessed on a clinical neuropsychological battery spanning multiple memory and non-memory domains (see Table 1). This was important to avoid previous criticisms of erroneous classification of MCI cases due to inadequate classification protocols. The aim of the screening stage was to identify those who met the criteria for MCI [5]. Performances were classified as subclinically impaired where the performance was more than 1.28SD (<10th percentile) below age- and/or education based norms in accordance with previously established protocols [6, 13, 21]. Classification of MCI subtype as single domain amnestic MCI (a-MCI), single domain non-amnestic MCI (na-MCI), multiple domain amnestic MCI (a-MCI+), or multiple domain non-amnestic MCI (na-MCI+) was based on the presence of one or more subclinical impairments to one or more cognitive domains [4, 6, 13, 21]. A total of 130 participants successfully complete pre-screening and classification screening. These participants composed the following groups: a-MCI (*n* = 24); na-MCI (*n* = 23); a-MCI+ (*n* = 27); na-MCI+ (*n* = 6); and healthy control (*n* = 50). Due to the statistical issues associated with analysing small samples, the na-MCI+ group were collapsed to form a larger na-MCI group. Prior to the reassessment of episodic memory, eight participants withdrew, four for personal reasons and four due to emerging chronic health issues. The final sample of 122 participants (male = 48) formed the following groups: a-MCI (*n* = 23); na-MCI (*n* = 25); a-MCI+ (*n* = 24); and healthy control (*n* = 50).

## 3. Materials

Participants were screened on a test battery (see Table 1) comprised of tests selected on the basis of excellent reliability and validity in clinical and subclinical populations. Follow-up episodic memory assessment (experimental) involved alternate tests of episodic memory to those used at screening assessment. Tests assessing both verbal and visual memory were included at screening and the experimental stages as research has shown that episodic memory deficits may manifest both verbally and/or visually in MCI [22]. The experimental protocol included the Paired Associates Learning test (PAL; [30]) and the The Rey Auditory Verbal Learning Test (RAVLT; [31]). The PAL is a subtest of the Cambridge Neuropsychological Test Automated Battery (CANTAB). It is a visual measure of episodic memory and learning and is sensitive to medial temporal lobe function [30]. The PAL has a demonstrated ability to accurately discriminate between individuals with AD and healthy controls as well as the capacity to predict future cognitive decline [32]. During the PAL, participants are presented with six white boxes that open up one at a time in random order. At trial one, the computer reveals two different patterns hidden in two separate boxes. The participant is required to recall the location of each pattern at the end of the presentation sequence. Correct detection of each pattern within the allocated ten attempts allows the participant to move on to the next phases where three, six, and eight patterns are hidden, respectively. Failure to recall the correct location of each pattern after 10 trials results in termination of the test. The selected outcome measures for the PAL were total errors at 6 and 8 shapes (adjusted), which report the number of errors made at each of the stages, respectively. These outcome measures were selected as they adjusts the total score for those participants who fail to meet criterion on an earlier trial and do not complete the entire PAL sequence [30]. The RAVLT is a verbal assessment of episodic memory and learning. The RAVLT consists of five consecutive learning trials of an auditory presentation of 15 item word list. Following each learning trial, participants recall as many of the 15 words in any order. After the fifth learning trial, a distracter list of 15 new words is presented followed by a recall trial. Following this, the participant is required to recall as many words possible from the initial list. Outcome measures used in the following analysis were RAVLT trial 5; RAVLT total (trials 1–5); and RAVLT delayed. The RAVLT has been found to be reliable in distinguishing between healthy controls and individuals with AD, as well as differentiating between various neurodegenerative conditions [33].

### 3.1. Procedure

Individual assessment sessions were conducted in a well-lit, well ventilated room and took approximately 90–120 minutes, including mandated rest breaks, to complete. Tasks assessing visual and verbal episodic memory were administered as part of a larger test battery examining the neuropsychological profile of MCI subtypes. Only results pertaining to episodic memory function were analysed for the present study. The CANTAB was administered on a laptop connected to an external 17 inch LCD touch screen monitor and response pad according to standard instructions. Participants sat approximately 50 cm from the touch screen with the response pad positioned 15 cm from the touch screen. 

## 4. Results

Results were analysed using SPSS for Windows (version 19.0). MANOVA was used to control for potential inflation of Type 1 error due to analysing data from multiple tests within the same domain (episodic memory). Significant multivariate results were followed with one-way ANOVAs and post hoc analyses. Games-Howell was considered the appropriate post hoc analysis due to unequal sample sizes and breaches of homogeneity of variance [34]. 

Demographic variables were assessed to examine any potential group differences that may act as potential confounds [31] (see Table 2). No group differences were detected in terms of age, education level, or HADS depression score. Group differences were detected on the WTAR with the a-MCI+ group having a significantly lower estimate of premorbid IQ compared to all groups. Group differences were also detected on HADS anxiety score however, due to insufficient power for the medium effect size evident a post hoc analysis failed to identify significant group differences, with a trend towards significance between the a-MCI+ and Control group (*P* = 0.068). Group differences in global cognitive function (DRS-2 score) were significant but in expected directions with the a-MCI+ having significantly lower scores than the control and na-MCI groups; and the a-MCI having significantly lower scores than the control group. While significant differences were found, no group had a mean DRS-2 score of clinical significance (all AEMSS ≥ 9). There was no significant difference in gender ratio across the four groups (*χ*_(3)_^2^ = 3.45, *P* = 0.327). 

A MANOVA identified significant group differences in episodic memory (PAL 6 shapes adjusted; PAL 8 shapes adjusted; RAVLT trial 5; RAVLT total; RAVLT delayed) (Pillai's trace = 0.260, *F*_(15,348)_ = 2.20, *P* = 0.006, power = 0.975, *n*_*p*_^2^ = 0.087). Group differences within each dependent variable were subsequently analysed by one-way ANOVA with post-hoc Games-Howell analysis. 

**Figure 1:**
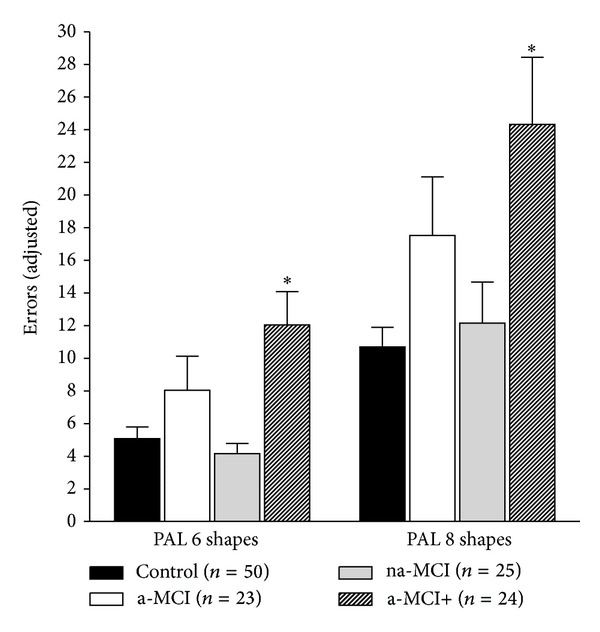
Group differences visual episodic memory (mean ± SEM).

Significant group differences were detected on PAL 6 shapes adjusted (*F*_(3,118)_ = 6.69, *P* < 0.001, power = 0.971, *n*_*p*_^2^ = 0.145) and PAL 8 shapes adjusted (*F*_(3,118)_ = 5.73, *P* = 0.001, power = 0.943, *n*_*p*_^2^ = 0.127). Post hoc analyses revealed that the a-MCI+ group made significantly more errors in attempting to recall the spatial location of six patterns compared to the na-MCI and control groups (Figure 1). At eight patterns, the a-MCI+ group made significantly more errors than the control group (Figure 1).

**Figure 2:**
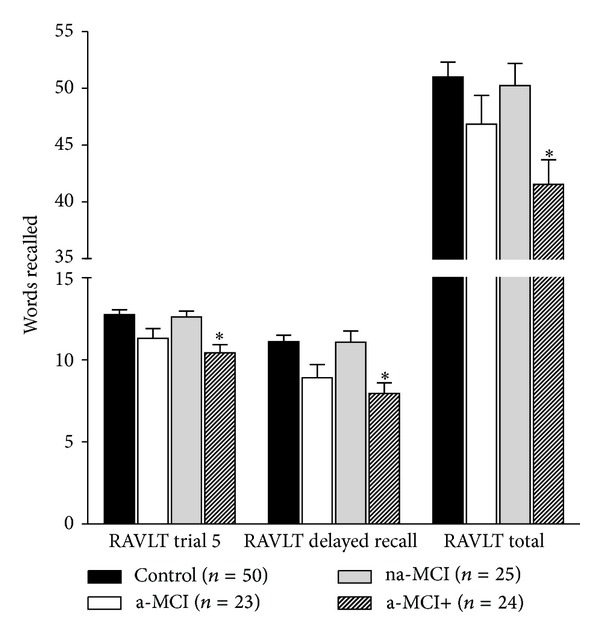
Group differences verbal episodic memory (mean ± SEM).

Significant group differences were detected on RAVLT trial 5 (*F*_(3,118)_ = 6.61, *P* < 0.001, power = .969, *n*_*p*_^2^ = .144); RAVLT total (*F*_(3,118)_ = 5.16, *P* = 0.002, power = .917, *n*_*p*_^2^ = .116); and RAVLT delay (*F*_(3,118)_ = 7.17, *P* < 0.001, power = 0.980, *n*_*p*_^2^ = 0.154). Post hoc analyses revealed that the a-MCI+ group recalled significantly less words on average than the na-MCI and control groups at trial 5 (Figure 2); across all RAVLT trials in total (Figure 2); and at the delayed recall stage (Figure 2).

## 5. Discussion

The results of the present study indicate that individuals identified as a-MCI+ from a comprehensive screening assessment display significantly lower performances on different measures of verbal and visual episodic memory compared to control participants or those classified as na-MCI. Specifically, the a-MCI+ group made significantly more errors when attempting to recall the spatial location of patterns (PAL 6 & 8 shapes adjusted). The a-MCI+ group also recorded the poorest performance on the final trial of a verbal learning task (RAVLT trial 5); lowest delayed verbal episodic memory recall (RAVLT delay); and poorest cumulative verbal learning across trials (RAVLT total). These results may seem unsurprising given that individuals within this group, by definition of their initial classification, scored at subclinical levels (<10th percentile) on at least one memory and one non-memory test at screening. That this group performed poorly on a different set of memory measures compared to those used at screening strongly suggests that circular reasoning in MCI research may be less problematic than previously suggested. 

While the a-MCI group appear to perform at an intermediate level between the a-MCI+ group and the control and na-MCI groups, these differences do not reach statistical significance. It could be argued that this is due to circular reasoning given that a new battery of memory tests was unable to identify significant group differences. However, a better explanation of these findings relates to stability. By definition, membership to the a-MCI subtype requires a single impaired performance on a single test of episodic memory. Previous research tracking MCI subtypes longitudinally suggests that the a-MCI profile is not only rare but highly unstable [21, 22, 35, 36]. That the a-MCI group performed at an intermediate level between the a-MCI+ group and the control and na-MCI groups may be a result of recovery of function of some individuals within this subtype. Therefore, it may be erroneous to conclude with certainty about circular reasoning in this group as performance differences could be confounded by false positive cases. 

The present study attempted to address the issue of circular reasoning in MCI research. The above data suggests that circular reasoning may be less of an issue given that the a-MCI+ subtype displays evidence of depressed verbal and visual episodic memory function on alternate tests conducted 9 months after initial assessment. However, it may be argued that the notion of circular reasoning within this context is flawed as it relies on the premise that MCI is stable. Research demonstrates that MCI is far from stable with consistent evidence that of recovery of function is common [6]. As a theoretical construct, if MCI is a precursor stage to dementia, it cannot be a stable entity. As a precursor to a neurodegenerative disease, one would expect that MCI should display a pattern of deterioration cognitive function(s) over time until the clinical stage of dementia is reached. As such, those identified as MCI should continue to display evidence of cognitive difficulties that have either remained stable or deteriorated over time. However, there should not be evidence recovery of function in genuine MCI cases as this would indicate erroneous classification within the MCI spectrum. 

Several factors warrant caution when interpreting the above data. First, the small sample size is likely to limit the generalisability of the present findings. Second, it could be argued that the issue of circular logic may have been better assessed by including a comparison group of individuals who were assessed with the same tests at screening and follow up. However, it is not possible to obtain two identical clinical groups for comparison. Further, by adopting this approach, it would be impossible to differentiate circularity effects from group differences and therefore confound the results. Third, it could be argued that circularity is inevitable unless there is complete independence between predictors and outcome measures [37]. The use of different tests tapping the same domains is likely to result in some degree of circularity as performance is likely to be highly correlated. However, this study represents one of the first attempts to formally investigate circular reasoning in MCI and has several strengths compared to previous research. All MCI cases were assessed using a comprehensive test battery rather than the conventional approach of using screening tests to classify MCI. In addition, both visual memory and verbal episodic memory were assessed as part of the screening classification and the follow up memory assessment. Previous research that has only examined verbal memory may have inadvertently missed classifying or misclassified cases where the memory impairment was visual in nature [22]. This study also represents one of the few that have not compromised the comprehensiveness of the screening protocol by using global measures in attempt to avoid circularity. 

Results of the present study show that when using different follow up tests, memory function remains compromised in individuals initially classified as a-MCI+. This suggests that circular reasoning in MCI research may be less of an issue than previously thought. Further, it implies that researchers are not justified in using broad global measures at screening to avoid the issue of circularity. Potential MCI cases should always be assessed with comprehensive test protocols that enhance diagnostic accuracy. However, future studies wanting to minimize the influence of circularity should adopt different classification and follow up protocols. More research is required as to how this procedure may impact the sensitivity and specificity of the MCI classification. 

## Figures and Tables

**Table 1:** Screening test battery used for MCI classification.

Test	Domain
Wechsler Test of Adult Reading (WTAR; [24])	Estimated premorbid IQ
Hospital Anxiety and Depression Scale (HADS; [25])	Clinical anxiety and depression symptoms
Mattis Dementia Rating Scale, 2nd edition (DRS-2; [26])	Global cognitive functioning
Rey Complex Figure Test (RCFT; [27])	Visual episodic memory
Logical Memory I & II (LM; [28] )	Verbal episodic memory
Digit Span (DSP; [29])	Immediate verbal memory span
Spatial Span (SSP; [28])	Immediate visual memory span
Letter-Number Sequencing (LNS; [29])	Working memory capacity
Stroop-Victoria version (Stroop; [27])	Executive functioning
Vocabulary (Vocab; [29])	Language function
Trail Making Test (TMT; [27])	Divided attention
Digit Symbol Coding (DSC; [29])	Sustained attention

**Table 2:** Group differences in Age, Education, Estimated Premorbid FSIQ, DRS-2, and HADS scores.

Measure *n*	a-MCI Mean (SD) 23	na-MCI Mean (SD) 25	a-MCI+ Mean (SD) 24	Control Mean (SD) 50	*P*	Post-hoc (at *P* < 0.05)	Effect size (*n*_*p*_^2^)	Power
Age	70.61 (7.99)	70.60 (5.97)	69.29 (6.42)	72.66 (6.52)	0.201		0.038	0.404
Education	14.43 (3.16)	15.04 (3.54)	12.46 (3.45)	14.20 (3.74)	0.072		0.057	0.585
WTAR (est. FSIQ)	110.22 (5.42)	110.24 (4.97)	103.33 (7.99)	110.38 (5.76)	<0.001	a-MCI+ < na-MCI, a-MCI, C	0.178	0.993
DRS-2 (AEMSS)	10.91 (2.17)	11.56 (1.89)	10.04 (1.97)	12.54 (2.14)	<0.001	a-MCI+ < na-MCI, C; a-MCI < C	0.183	0.994
HADS A	5.39 (2.79)	5.00 (2.68)	6.88 (3.71)	4.72 (2.56)	0.028	*Insufficient power *	0.074	0.719
HADS D	3.04 (2.34)	2.52 (2.02)	3.38 (2.46)	2.72 (2.33)	0.558		0.017	0.193

WTAR: Wechsler Test of Adult Reading; est FSIQ: estimated Full Scale Intelligence Quotient; DRS-2: Dementia Rating Scale-2 (Age and Education corrected); HADS: Hospital Anxiety and Depression Scale (A: Anxiety score; D: Depression score); C: control.
